# Stealthy data integrity attack identification in smart grid networks utilizing deep denoising autoencoder

**DOI:** 10.1016/j.heliyon.2024.e38470

**Published:** 2024-09-27

**Authors:** Anila Kousar, Saeed Ahmed, Abdullah Altamimi, Su Min Kim, Zafar A. Khan

**Affiliations:** a Department of Electrical Engineering, Mirpur University of Science and Technology (MUST), Mirpur AJK, 10250, Pakistan; b Department of Electrical Engineering, College of Engineering, Majmaah University, Al-Majmaah, 11952, Saudi Arabia; c Engineering and Applied Science Research Center, Majmaah University, Al-Majmaah, 11952, Saudi Arabia; d Department of Electronics Engineering, Tech University of Korea (TU Korea), Siheung-si, 15073 237, Gyeonggi-do, South Korea

**Keywords:** Cyber-physical systems, Cyber assaults, Smart grids, State estimation, Deep denoising autoencoder, Machine learning

## Abstract

Smart grids arose as the largest cyber-physical systems with the integration of sophisticated control, computing, and state-of-the-art communications. Like all cyber-physical systems, the smart grids are vulnerable to malicious cyber assaults due to their enormous dependency on communication networks. Various machine learning-based schemes are being investigated in the industry and academia to develop robust defense mechanisms to counter cyber assaults. However, the curse of high dimensionality, which increases with the escalating evolution of an electric power system, infringes upon the efficiency of machine learning models employed to detect such assaults. To this end, this paper proposes a deep denoising autoencoder (DAE)-based framework for dimensionality reduction that learns salient feature representation for high-dimensional, multi-variant smart grid measurement data collected from the smart grids. The latent space apprehended by DAE is then fed to binary support vector machine (SVM) to determine the assaulted data. Various standard IEEE test cases are employed in simulations. The results show that the proposed scheme learns more robust features that reveal the nonlinear properties exhibited in the smart grid measurements, further leading to improved detection accuracy of the classifier as compared to existing approaches.

## 1 Introduction

With the integration of state-of-the-art communication, computing and advanced control, smart grids (SGs) have developed into the most sophisticated cyber-physical system (CPS). However, SGs are more susceptible to multiple types of cyber-assaults. [1], [2], [3], [4], [5], [6], [7]. The stealthy data integrity assault (SDIA), is considered capable of decieving the bad data detector (BDD), which is traditionally embedded in the power control center (PCC), to identify the assaulted data [8]. Multiple studies are being carried out in the industry and academia to investigate the cyber threats to SGs and design countering mechanisms.

Lately, artificial intelligence-based approaches have earned the attention of researchers to detect flaws, intrusions, and anomalies in many domains of various CPSs. Machine learning (ML) is discovering its applications in the security of SGs as well. Numerous studies utilized a variety of ML-based techniques to identify the cyber-assaults on the physical layer of SG communication networks [9], [10], [11]. However, it becomes complicated to ML models when the SG dataset expands with the growing dimensions of power system resulting in more splendid intricacy and complexity in computations and increased time consumption for model training [12], [13], [14]. Likewise, the efficiency of the ML models in classifying the assailed SG data from the normal data is greatly affected by the curse of dimensionality [14]. Implementation of dimension reduction (DR) schemes results in feature space of high relevance, thus, further improving the generalization capability of the ML algorithms.

Typically, the DR techniques are categorized as 1) Feature extraction (FE) and Feature selection (FS). FS techniques select the most relevant features while eliminating the less significant features or variables to obtain a space that is in actual a subset of the original feature space. However, neglecting some features may lead to a decrease in overall effectiveness. [15], [10], [16]. This issue is addressed by the FE techniques, where the high-dimensional feature space undergoes a projection onto a lower-dimensional space using either linear or non-linear techniques while keeping intact the original properties of the data. Furthermore, the FE-based techniques are more prolific in tackling the assailed or corrupted data. Traditionally, FE utilizes linear discriminant analysis and principal component analysis as feature extraction techniques [17], [18], [19]. However, these tools are inefficient when it comes to extracting the distinctive features of datasets that exhibit non-linear patterns, such as SG data. Recent studies reveal that a deep denoising autoencoder (DAE) can effectively model the non-linear properties of the multi-variant data [16], [20], [21]. The DAE is a variant of autoencoder(AE) and, it reconstructs the input data at the output employing non-linear transformation through the series of hidden layers. Hence, while the non-linear representation is learned from the hidden layer, the dimensions are also reduced.

Contrary to previous approaches, this paper proposes a DAE-based DR scheme for the SG data set. Next, we employed the ML-based binary SVM scheme for detection of cyber stealthy data integrity attack (SDIA) in the dataset. The comparison of the presented scheme with the existing dimensionality reduction methods shows that the proposed technique results in a robust latent-space representation code that is more effective in detecting the SDIA. We summarise the contributions of the paper as follows:

• We examine an intelligently crafted SDIA, employed to compromise the SG measurements and investigate the increase in the dimensions with the increasing size of the data and analyze the impact of the dimensions in classifying the assaulted data.
• Feature selection techniques are inefficient to mine characteristics of nonlinear dataset such as SG measurements. Therefore, we employed feature extraction-based DAE scheme to capture salient feature representation for high-dimensional, multi-variant smart grid measurement data collected from the smart grids, which ultimately reduces the computational burden to identify SDIA.
• The SDIA detection in SG data is a binary classification challenge, specifically involving the classification of data samples into two categories: attacked and non-attacked. For this we used Support Vector Machine algorithm. The latent space apprehended by DAE is then fed to SVM binary classifier as input to determine the assaulted data.
• To determine the performance efficiency of DAE-based DR and SVM-based SDIA detection scheme, we used standard IEEE 14-bus, 39-bus, and 57-bus systems as test cases. The comparison results with existing DR and anomaly detection schemes showed that the proposed scheme outperforms others in learning robust features that reveal the nonlinear properties exhibited in the smart grid measurements, further leading to improved detection accuracy of the classifier.

The structure of remaining paper is as: Section 2 explains the SDIA and components of a smart power system. Section 3 furnishes working principle of the denoising autoencoder while the proposed DAE-based DR and SVM-based SDIA detection schemes is presented in section 4. Simulation results are furnished in section 5. Lastly, conclusion is drawn in section 6.

## 2 Smart grid operations and stealthy data integrity attack

Power plants are connected to the consumers via power transmission systems across a vast geographic region. Redundant lines are employed to secure the routing of the power and ensure the economy of the transmission route. A fast and reliable communication network establishes link between power system entities and PCC for effective monitoring and control.

### 2.1 State estimation

Bi-directional remote terminal units, comprising sensors and actuators, are utilized in the SGs for monitoring and control. At the PCC, the state variables (SVs), $\phi ={\left[\phi_{1},\phi_{2},...,\phi_{p}\right]}^{T}$, are estimated utilizing the sensor-collected measurements, $Z_{s}={\left[z_{1},z_{2},...,z_{q}\right]}^{T}$, given in equation (1) *p* and *q* are positive numbers, and $\phi_{r},z_{s}\in \mathrm{IR}$ for r=1,2,...,p and s=1,2,...,q. To be more precise, in an alternating current or nonlinear model, these SVs are linked to the sensor-collected measurements as given below:

(1) $$Z_{s}=h(\phi )+\psi ,$$

where *h* describes the nonlinear association between $Z_{s}$ and *ϕ*, and *ψ* is an error vector or a Gaussian measurement noise, $\psi ={\left[\psi_{1},\psi_{2},...,\psi_{m}\right]}^{T}$.

Presuming that at each bus, the magnitude of voltage remains close to rated value, equation (1) can be simplified employing the following direct current model as given below in equation (2):

(2) $$Z_{s}=H\phi +\psi .$$

where *H* is termed the Jacobian matrix, and estimated in equation (3) as [22], [23]:

(3) $$H={\frac{\partial h(\phi )}{\partial \phi }|}_{\phi =0},$$

In order to determine the estimation, $\overset{ˆ}{\phi }$, of the state vector *ϕ* that fits the most to the sensor-collected values, three most widely used statistical criteria are employed: weighted least squares, maximum likelihood, and minimum variance [24]. These criteria result in a similar voltage phase estimate considering normally distributed sensor error with a zero mean, as given below in equation (4):

(4) $$\overset{ˆ}{W}={\left(H^{T}WH\right)}^{-1}H^{T}WZ_{s}=\Delta Z_{s},$$

where $W=(H^{T}{\mathrm{WH})}^{-1}H^{T}W$, and W is a diagonal matrix presented in equation (5) as:

(5) $$W=\left[\begin{array}{ccccc} \sigma_{1}^{-2}\; & 0\; & 0\; & 0\; & 0\\ 0\; & .\; & 0\; & 0\; & 0\\ 0\; & 0\; & .\; & 0\; & 0\\ 0\; & 0\; & 0\; & .\; & 0\\ 0\; & 0\; & 0\; & 0\; & \sigma_{m}^{-2} \end{array}\right].$$

### 2.2 Conventional bad data detection

Sensor malfunctioning, hostile cyber-assaults, and wireless medium noise may corrupt the sensor measures. In normal circumstances, measurements collected by the sensors make an approximation of the SVs in close proximity to the actual values. On the other hand, an SDIA may alter state values rendering a dichotomy between the assaulted and unassaulted values. Conventional electrical networks utilize a detector based on residual measurements to detect anomalies in the $Z_{s}$, [25]. The dissimilarity between the estimated measurements, ${\overset{ˆ}{Z}}_{s}$, and sensor-gathered measurements, $Z_{s}$, is termed residual, *R*, and is calculated at the PCC using equation (6) given below:

(6) $$R=Z_{s}-\overset{ˆ}{Z_{s}}=Z_{s}-H\overset{ˆ}{\phi }.$$

Then, $\left\|Z_{s}-H\overset{ˆ}{\phi }\right\|$ is scrutinized with a threshold, *λ* [26], to identify the bad data. Consequently, the supposition of being unattacked is abode if we have satisfied the condition given in equation (7).

(7) $$\underset{i}{\max}\;\left|R_{i}\right|<\lambda ,$$

otherwise, an alarm signifying presence of noisy data is generated,

### 2.3 Stealthy data integrity attack: basic principle

An intelligent hacker can attempt to compromise the wireless channels to infuse false values in sensor-collected measurements, even with a partial knowledge of the power network hierarchy. The hacker may form an assault vector, $\varepsilon =\left[\varepsilon_{1}+\varepsilon_{2},...,\varepsilon_{m}\right]$, to dupe the BDD [9]. Let $Z_{\varepsilon }=Z_{s}+\varepsilon$, be comprising the attacked data. In attack vector *ε*, the hacker may choose a random non-zero member. Thus, the *i*th non-zero element, $\varepsilon_{i}$, of attack vector *ε*, permits the hacker to replace the *i*-*th* sensor data, $Z_{s_{i}}$, with a fabricated data: $Z_{s_{i}}+\varepsilon_{i}$.

Typically, the BDD employs the calculation of the $L_{2}-\mathrm{Norm}$ of *R* as a means to determine the presence of corrupted data. Nevertheless, in the event that the hacker devises the attack vector *ε*, defined as ε=Hc, where c represents a non-zero vector with a length of n, it is possible for the measurement vector containing the attacks $\left(Z_{\varepsilon }\right)$ to evade the BDD, as long as the measurement vector with normal values is able to pass undetected. Let ${\overset{ˆ}{\phi }}_{\varepsilon }$ represent an estimation of state variables using compromised sensor measurements $Z_{\varepsilon }$, such that we get equation (8) as given below:

(8) $${\overset{ˆ}{\phi }}_{\varepsilon }=\Delta Z_{s}+\Delta \varepsilon =\overset{ˆ}{\varepsilon }+\Delta Hc=\overset{ˆ}{\phi }+c.$$

Thus, the $L_{2}$ norm of attacked measurements residual $R_{\varepsilon }$ is obtained as presented in equation (9)

(9) $$\begin{array}{c} {\left\|R_{\varepsilon }\right\|}_{2}={\left\|Z_{\varepsilon }-H{\overset{ˆ}{\phi }}_{\varepsilon }\right\|}_{2}\\ =\left\|(Z_{s}+\varepsilon )-H({\overset{ˆ}{\phi }}_{a}+c)\right\|\\ ={\left\|\left(Z_{s}-H\overset{ˆ}{\phi }\right)+\left(\varepsilon -Hc\right)\right\|}_{2}={\left\|\left(Z_{s}-H\overset{ˆ}{\phi }\right)\right\|}_{2}\\ ={\left\|R\right\|}_{2}<\lambda . \end{array}$$

### 2.4 SDIA attack model

The adversary can intelligently evade the operator working at the PCC and elude the BDD by designing an attack which manipulates one or multiple values in the measurements. Generally, the SDIAs are categorized as load change assaults and load redistribution assaults [27], [28], [29]. During an SDIA assault, the adversary infuses a fabricated value in the data collected by sensors by changing the real power flows and power injection. This manipulation is carried out with the intention of projecting a desired alteration in the state variables. For example, to alter the SV $z_{2}$ by inserting a corruption of −10%, an attack vector *c* of (1×(p−1)) can be crafted by taking into account the equation (10) given below:

(10) $$c=[-0.10z_{2},0,...,0].$$

Employing the state vector $\phi_{\varepsilon }=\overset{ˆ}{Z_{s}}+c$ and power flow equations, the assailed measurements are calculated as depicted in equation (11):

(11) $$Z_{\varepsilon }=H\phi_{\varepsilon }+\psi .$$

## 3 Working principal of denoising autoencoder (DAE) scheme

**Figure 1:**
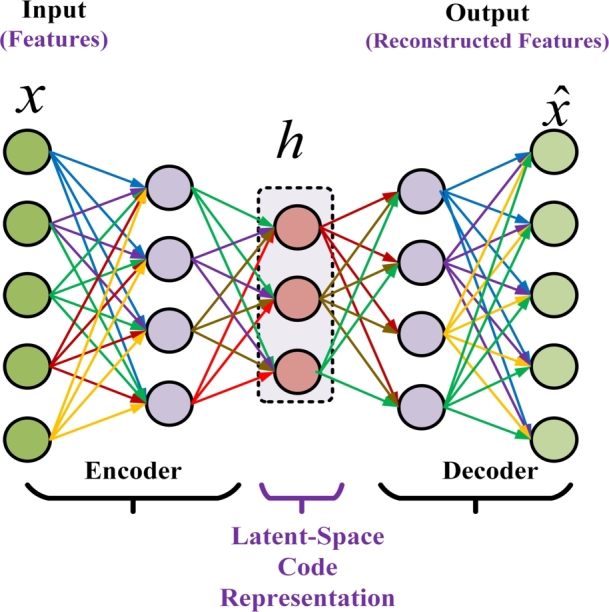
Deep denoising autoencoder model.

High dimensional space results in a high cost increasing computational complexity and large time consumption to train ML models. In order to address the issue of high dimensionality in the SG data, referred to as the state estimation-measurement features (SE-MF) data set in this study, we introduce a dimensionality reduction scheme based on deep denoising autoencoders. Next, we explain the foundational principle of the suggested scheme. The DAE model is shown in Fig. 1

Autoencoder (AE) is a unique distinct form of perfectly connected feed-forward neural network, characterized by having an equal number of inputs and outputs. Mandating no-label, the AE is trained in an unsupervised way. In this paper, to grasp the hidden nonlinear correlations in a complex multivariate SE-MF data set, we use a lately conceived variant of AE, the denoising autoencoder (DAE) [30]. As an extension of AE, the DAE is robust in handling the assailed or corrupted data.

The rudimentary idea of the DAE is to reconstruct original data from bad assaulted input [20]. Unlike the AE, where merely identity mapping is learned betwixt the revamped output and original input dataset, the DAE has the capability to capture more explanatory and informative latent-space imprints and pick a vigorous representation from unprocessed, or assaulted measurements. Two basic preferences for corruption addition are additive Gaussian noise (GDAE) and zero-masking noise (ZDAE) [20], [30].

Like AE, the DAE consists of three main components: the encoder, latent-space-code, and the decoder. Given the input *x*, the encoder typically employs nonlinear mapping, as given below, to translate the corrupted or flawed input data $\overset{˜}{x}$ into a latent-space model *h* expressed in equation (12), instead of using the original input data *x*.

(12) $$h=f\left(w_{1}x+b\right),$$

where $w_{1}\in IR^{m\times n}$ is the weight matrix, and $b\in IR^{m}$ is optimized tor with *m* nodes in latent space. At output layer, the decoder unravels, utilizing nonlinear transformation, the latent space into a reconstructed vector, $\overset{ˆ}{x}$, as demonstrated in equation (13):

(13) $$\overset{ˆ}{x}=g\left(w_{1}x+c\right),$$

To attain a high apprehension performance, we utilized the tied weights as $w_{1}=w_{2}^{T}$ [30]. The error in reconstruction is calculated for a given input training dataset, ${\left\{x_{i}\right\}}_{i=1}^{m}$, as follows: $\sum_{i=1}^{m}{\left\|x_{i}-{\overset{ˆ}{x}}_{i}\right\|}^{2}$. The objective in training is to discover optimal parameters, $\psi =\left\{W_{1},b,c\right\}$ that can reduce reconstruction inaccuracy in the following manner:

(14) $$\underset{\psi }{\min}\;\sum_{i=1}^{m}{\left\|x_{i}-{\overset{ˆ}{x}}_{i}\right\|}^{2}.$$

Equation (14) reveals that the error in reconstruction is the gap betwixt the reconstructed output and the original sensor data rather the assaulted data. Specifically, the DAE is trained to generate an output that closely resembles the original input *x*, even in cases where the input is corrupted or altered, $\overset{˜}{x}$.

## 4 Proposed DAE-based dimension reduction and SVM-based attack detection scheme (DAE+SVM)

**Figure 2:**
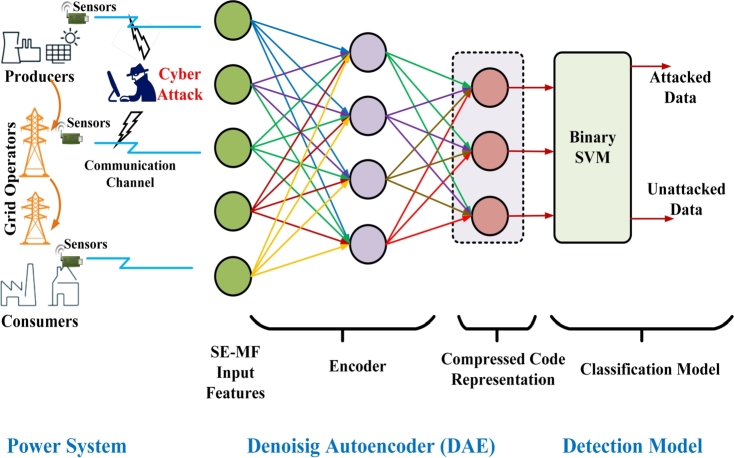
Deep denoising autoencoder-based feature extraction for stealthy attack detection in smart grid measurement data set.

Power transmission networks transmit the generated electric power to the load. The sensors employed at various entities of the power network collect and forward the SE-MF data over wireless channels. A hacker may insert biased values into the transmitted data to compromise the integrity of the data. After receiving the data at the PCC, the deep denoising AE model endeavors to obtain a latent space representation. Next, the compressed code, latent space, is given to the SVM-based model in oder to identify the SDIA. Layout of the proposed scheme is presented in Fig. 2. Note that the compressed code representation is basically projection of high dimension space onto low dimensional space which leads to lower the computational burden for anomaly detection.

To train the DAE model, the GDAE and ZDAE are the two familiar methods of injecting corruption to the system [31], [30]. In the ZDAE scheme, a few data features from all samples are randomly switched to zero with a probability *v*. Zero masking is identical to blank sensor measurements due to noise or adversary attacks. This paper introduces a novel approach, referred to as EDAE, for incorporating corruption into data. The corruption is introduced by considering a Gaussian distribution with mean and variance determined through the analysis of the SE-MF data. The subsequent section explains the corruption or noise addition mechanism utilizing EDAE.

### 4.1 Proposed EDAE corruption addition scheme

A well tained DAE model is significantly important to obtain a latent space representation of the high dimension. During the training phase, corruption or noise is introduced to the training dataset denoted as $X=\left\{x_{1},x_{2},...,x_{m}\right\}$, where *m* represents the number of data samples. Each data sample $x_{i}$ belongs to the set *X*, where *i* ranges from 1 to *m*. The variable $x_{i}$ represents a sample that contains *n* features. Each feature $fe_{ij}$ is an element of the set $x_{i}=\left\{fe_{i1},fe_{i2},...,fe_{in}\right\}$. The noise *δ* that has been introduced follows a normal distribution. The distribution N(μ,ν) is considered, where *μ* represents a vector containing the mean values, $\mu =\left\{\mu_{1},\mu_{2},...,\mu_{n}\right\}$, which is determined as $\mu_{j}=\frac{1}{m}\sum_{i=1}^{m}fe_{ij}$. The variance vector, denoted as $\nu =\left\{\nu_{1},\nu_{2},...,\nu_{n}\right\}$, is defined as the collection of variances for each variable. Each element $\nu_{j}$ of the variance vector is calculated as the average squared difference between the observed value $\mu_{j}$ and the corresponding estimated value $fe_{ij}$, summed over all *m* observations. Subsequently, the training data are yielded as $X_{0}=X+\delta$, to train the DAE model.

### 4.2 Support vector machine-based detection of the stealthy data integrity attack

After the DAE model apprehends the robust latent-space representation *h*, given in equation (14), it is fed as input to SVM for classification as shown in the Fig. 1. The SDIA detection in SG data is a binary classification challenge, specifically involving the classification of data samples into two categories: attacked and non-attacked. The Support Vector Machine algorithm effectively addresses the task of binary classification by identifying the hyperplane with the maximum margin that separates the two categories within the feature space of the training dataset [32]. In order to differentiate between the labels of test samples belonging to the attacked samples and the non-attacked data, the sign of the hyperplane function is utilized. The study utilizes the Gaussian radial basis function as the chosen kernel function. The SVM algorithm condenses to address the optimization issue by utilizing the Lagrange multipliers as illustrated by equation (15):

(15) $$\begin{array}{c} \arg\max\left\{\sum \alpha_{i}-\frac{1}{2}\sum_{i=1}^{N}\sum_{j=1}^{N}\alpha_{i}\alpha_{j}y_{i}y_{j}K(x_{i},x_{j})\right\},\\ s.t\sum_{i=1}^{N}\alpha_{i}y_{j}=0,\;0\leq \alpha_{i}\leq C\;\forall i=1,2,...,N. \end{array}$$

In equation (15), the samples from the training SE-MF data are represented by $x_{i}$ and $y_{i}$, and the Lagrangian multipliers are denoted by $\alpha_{i}$'s. The variable *C* is utilized as a penalty parameter in order to regulate the inference performance of the SVM classifier, and its value can be adjusted accordingly. The kernel function, denoted as, $K(x_{i},x_{j})$, is derived from Mercer's Theorem [33]. The related decision or classification function associated with the SVM is retrieved as shown in equation (16):

(16) $$\begin{array}{c} F(x)=\mathrm{sgn}\;\left\{f(x)\right\},\\ \mathrm{where}f(x)=\sum_{i=1}^{N}\alpha_{i}^{⁎}y_{i}^{⁎}K(x_{i}^{⁎},x)+b. \end{array}$$

The function f(x) ranges from −∞ to +∞ and, it represents the signed distance of the unspecified sample from the decision boundary. A positive value of the decision for a class implies that *x* is an unassailed data sample. On the other hand, a negative value suggests otherwise [34]. The RBF kernel is commonly employed when dealing with linearly non-separable data and, it is determined using equation (17):

(17) $$k(x_{i},x_{j})=\exp\left(-\omega {\left\|x_{i}-x_{j}\right\|}^{2}\right),$$

where $\omega =\frac{1}{2\sigma^{2}}$ and *σ* are adaptable parameters to be chosen cautiously. For smaller values of *σ*, the exponential is linear and, its non-linear potential reduces with higher-dimensional projection. For large *σ* values, due to lack of regularization, the decision boundary becomes super sensitive.

## 5 Simulations

In this section, first, we explain the SE-MF data generation, SDIA scenarios, and simulation parameters for DAE and SVM models. Next, the performance evaluation for the proposed DAE-based dimension reduction and SVM-based SDIA detection is presented. Table 1, Table 2 show the simulation parameters employed for the tuning of DAE and SVM models, respectively.

**Table 1:** Simulation parameters for deep denoising autoencoder (DAE)-based feature extraction model.

Parameters	Standard IEEE System		
	Bus-14	Bus-39	Bus-57
Input/ Output Nodes	53	130	216
Complete Data Samples	200,000	200,000	200,000
Train Data Samples	100,000	100,000	100,000
Test Data Samples	100,000	100,000	100,000
Latent Space size	34	75	140
DAE Layers	3	2	2
Error Estimator/Optimizer	Mean Square Error		
Batch Size	1		
Epochs	30		
Normalization	Min-Max

**Table 2:** Five finest-reconstructed features (Static-attack affecting 20% of SE-MF data features).

EDAE-Based Reconstruction Scheme (IEEE 14-bus System)					
Feature Number	38	46	49	24	18
Feature Value	−42	−17.574	−6.8649	8.09	50.4
Reconstructed Value	−4.20×10^1^	−1.76×10^1^	−6.86×10^0^	8.09×10^0^	5.04×10^1^
Error	5.18×10^−5^	6.14×10^−5^	6.47×10^−5^	8.01×10^−5^	0.00010462
Error Ratio	6.75×10^−7^	7.19×10^−6^	1.12×10^−6^	4.24×10^−6^	6.09×10^−6^

### 5.1 SE-MF data generation

We employed numerous standard test cases from the power transmission network such as IEEE standard bus systems namely 14 (Bus-14), 39 (Bus-39), and 57 (Bus-57) for validating effectiveness of the proposed solution. To design these IEEE test cases and specifically, the *H* matrix, we used the Matpower 7.0 toolbox [35] using DC power flow analysis. For a power transmission system consisting of *J* buses, the state vector *ϕ*] comprises of (J−1) bus voltage phase angles. The measurement features in the SE-MF data set include active power flows in the transmission lines and active power injections into the buses. We assumed stochastic loads with uniform load distributions similar to those in [36] to execute an impartial comparison with real-world scenarios.

### 5.2 Attack scenarios

As discussed earlier, the foremost aim of SDIA is to corrupt various or single system states. Assuming the hacker, fully cognizant of the power transmission network topology, launches the assault considering the model explained in [37]. The attack design approach is presented in Section 2. We evaluated various assault strategies in the simulations as described in the following:

**Scenario 1-Static Assault**: In this assault scenario, the adversary is presumed dormant with access to limited meters or sensors. Therefore, the adversary can only launch a static attack, i.e., Static features of the data samples are affected during the attack.

**Scenario 2–Nomadic Attack**: In the nomadic scenario, the hacker is assumed to change its location and randomly access various sensors or meters. While initiating a nomadic attack, the attacker randomly inserts the fabrications into the data samples. This type of attack is more emphatic and difficult to classify.

In the earlier mentioned attack scenarios, 40% and 20% of the features in the SE-MF data set were attacked, respectively.

### 5.3 Tuning the parameters for denoising autoencoder

We used IEEE power network test cases to generate the synthetic SE-MF data. With the growing size of the power network, the dimensions of the SE-MF dataset increase. For different power system test cases, the number of input nodes is different for the DAE model. A well-trained DAE model revamps the output, identical to the original input; hence, the number of output and input nodes is the same. The DAE model was trained using 50% of the generated SE-MF data. Fine-tuned parameters are selected considering optimal reconstructed data in each standard IEEE test bus case. Table 1 shows the simulation parameters. We adopted eclectic corruption addition schemes such as EDAE, ZDAE, and GDAE, to expand our analysis viewing account the expanded attack margin.

### 5.4 Tuning the parameters for support vector machine

The extracted compressed code latent space is engaged as input to the SVM to identify samples in the SE-MF dataset. As shown in Table 14, using 4-fold cross-validation with RBF, we used 75% data (out of 1000 SE-MF data samples) for training the SVM model. Next, to test the accuracy of the fitted decision boundary, the rest of the data is utilized. A meticulous selection of penalty parameters *C* and *σ* enhances the classification performance of the SVM model. The parameter *C* determines the precision of the decision boundary between the normal and the attacked classes, while the parameter *σ* specifies the impact of a single training sample. Optimal values of *σ* and *C* result in a minimum classification error. Therefore, to obtain optimal values of *σ* and *C*, we search from an extensive range of values between $10^{-5.1}$ and $10^{+5.1}$ utilizing Bayesian optimization.

### 5.5 Performance evaluation of DAE-based dimension reduction

The detection accuracy of the classification model mainly depends on the optimality of the latent space code obtained from the DAE model or, in other words, the accuracy of the reconstructed features by the DAE is significant for extracting a suitably robust latent space. One way to evaluate the fineness of the latent space is to examine the accuracy of the reconstructed features at the output layer of the DAE model. Therefore, first, we show the accuracy of the DAE-based reconstructed features. In this paper, the DAE model is constructed considering standard IEEE 14-, 39-, 57-bus systems yet, we show results exclusively from the standard IEEE 14- and 39-bus systems to limit the length of the paper.

Table 2, Table 3, Table 4, Table 5, Table 6, Table 7, Table 8, Table 9, Table 10, Table 11, Table 12, Table 13 show the original feature values, DAE-based reconstructed feature values, the reconstruction error, and the reconstruction error ratio in the revamped SE-MF data set. It can be seen from Table 1 that 14-bus system has 53 features or input nodes and, due to space constraints, it is not viable to show all the reconstructed features of the data. Therefore, few finest-reconstructed data features are shown for the proposed EDAE-, and existing ZDAE- and GDAE-based data reconstruction schemes. The static and nomadic attack scenarios are analyzed in the simulations with 20% and 40% attacked features in the SE-MF dataset.

**Table 3:** Five finest-reconstructed features (Static-attack affecting 40% of SE-MF data features).

EDAE-Based Reconstruction Scheme (IEEE 14-bus System)					
Feature Number	16	24	18	36	44
Feature Value	71.248	6.7785	50.363	−8.1342	−71.248
Reconstructed Value	7.12×10^1^	6.78×10^0^	5.04×10^1^	−8.13×10^0^	−7.12×10^1^
Error	6.05×10^−6^	2.60×10^−5^	3.40×10^−5^	5.84×10^−5^	7.95×10^−5^
Error Ratio	8.50×10^−8^	3.84×10^−6^	6.75×10^−7^	7.19×10^−6^	1.12×10^−6^

**Table 4:** Five finest-reconstructed features (Static-attack affecting 20% of SE-MF data features).

ZDAE-Based Reconstruction Scheme (IEEE 14-bus System)					
Feature Number	29	41	9	35	15
Feature Value	−5.7354	−29.115	−10.608	−73.443	73.443
Reconstructed Value	−5.729508	−29.10641	−10.59362	−73.42702	73.460947
Error	0.005892	0.008590	0.014379	0.015984	0.017947
Error Ratio	0.001028	0.000295	0.001355	0.000218	0.000244

**Table 5:** Five finest-reconstructed features (Static-attack affecting 40% of SE-MF data features).

ZDAE-Based Reconstruction Scheme (IEEE 14-bus System)					
Feature Number	35	15	32	52	19
Feature Value	−73.47	88.164	1.7611	−1.4676	−24.31
Reconstructed Value	−7.35×10^1^	8.82×10^1^	1.77×10^0^	−1.45×10^0^	−2.43×10^1^
Error	0.009265	0.009674	0.010849	0.013502	0.021026
Error Ratio	1.26×10^−4^	1.10×10^−4^	6.16×10^−3^	9.20×10^−3^	8.65×10^−4^

**Table 6:** Five finest-reconstructed features (Static-attack affecting 20% of SE-MF data features).

GDAE-Based Reconstruction Scheme (IEEE 14-bus System)					
Feature Number	47	12	27	18	25
Feature Value	−28.447	−15.038	28.447	−24.427	17.551
Reconstructed Value	−2.84×10^1^	−1.50×10^1^	2.84×10^1^	−2.44×10^1^	1.76×10^1^
Error	0.0002416	0.0003304	0.0006857	0.0007730	0.00110307
Error Ratio	8.50×10^−6^	2.20×10^−5^	2.41×10^−5^	3.16×10^−5^	6.28×10^−5^

**Table 7:** Five finest-reconstructed features (Static-attack affecting 40% of SE-MF data features).

GDAE-Based Reconstruction Scheme (IEEE 14-bus System)					
Feature Number	17	28	22	48	37
Feature Value	41.969	5.7354	43.655	−5.7354	−41.969
Reconstructed Value	41.972399	5.738713	43.65632	−5.734781	−41.96699
Error	0.104959	0.1311273	0.1450200	0.1598769	0.131724
Error Ratio	8.1006×10^−5^	0.0005	3.033×10^−5^	−0.000108	−4.79×10^−5^

**Table 8:** Five finest-reconstructed features (Nomadic-attack affecting 20% of SE-MF data features).

EDAE-Based Reconstruction Scheme (IEEE 14-bus System)					
Feature Number	25	41	24	50	21
Feature Value	1.76×10^1^	−1.69×10^1^	7.73×10^0^	2.99×10^0^	1.69×10^1^
Reconstructed Value	1.76×10^1^	−1.69×10^1^	7.73×10^0^	2.99×10^0^	1.69×10^1^
Error	2.42×10^−5^	3.72×10^−5^	0.00012197	0.0001456	0.00022623
Error Ratio	1.38×10^−6^	2.20×10^−6^	1.58×10^−5^	4.88×10^−5^	1.34×10^−5^

**Table 9:** Five finest-reconstructed features (Nomadic-attack affecting 40% of SE-MF data features).

EDAE-Based Reconstruction Scheme (IEEE 14-bus System)					
Feature Number	29	32	43	40	14
Feature Value	5.6798	5.2682	−6.7198	−29.111	73.554
Reconstructed Value	5.68×10^0^	5.27×10^0^	−6.72×10^0^	−2.91×10^1^	7.36×10^1^
Error	6.26×10^−5^	0.00022425	0.00040654	0.00049085	0.00053442
Error Ratio	1.10×10^−5^	4.26×10^−5^	6.05×10^−5^	1.69×10^−5^	7.27×10^−6^

**Table 10:** Five finest-reconstructed features (Nomadic-attack affecting 20% of SE-MF data features).

ZDAE-Based Reconstruction Scheme (IEEE 14-bus System)					
Feature Number	42	44	14	41	21
Feature Value	−43.836	−7.734	73.554	−16.888	16.888
Reconstructed Value	−43.836	−7.73421	73.555749	−16.88659	16.88984
Error	0.001948	0.0019416	0.0018449	0.0014046	0.0018449
Error Ratio	4.45×10^−5^	0.000251	2.38×10^−5^	8.32×10^−5^	0.000109

**Table 11:** Five finest-reconstructed features (Nomadic-attack affecting 40% of SE-MF data features).

ZDAE-Based Reconstruction Scheme (IEEE 14-bus System)					
Feature Number	18	14	38	44	24
Feature Value	−24.313	73.554	24.313	−7.734	7.734
Reconstructed Value	−24.3109	73.55617	24.316736	−7.729402	7.739212
Error	0.002088	0.002166	0.003736	0.004598	0.005212
Error Ratio	8.59×10^−5^	2.945×10^−5^	1.54×10^−4^	5.95×10^−4^	6.74×10^−4^

**Table 12:** Five finest-reconstructed features (Nomadic-attack affecting 20% of SE-MF data features).

GDAE-Based Reconstruction Scheme (IEEE 14-bus System)					
Feature Number	21	20	24	30	4
Feature Value	16.888	29.111	6.7198	9.9661	−11.794
Reconstructed Value	16.8896597	29.112748	6.7216329	9.9679635	−11.791911
Error	0.0016597	0.0017482	0.001833	0.001863	0.0020888
Error Ratio	9.83×10^−5^	6.01×10^−5^	2.73×10^−4^	1.86×10^−4^	1.77×10^−4^

**Table 13:** Five finest-reconstructed features (Nomadic-attack affecting 40% of SE-MF data features).

GDAE-Based Reconstruction Scheme (IEEE 14-bus System)					
Feature Number	33	13	42	14	22
Feature Value	−153.53	153.53	-43.836	73.554	43.836
Reconstructed Value	−153.52586	153.532541	−43.83366	73.555113	43.838355
Error	0.00414493	0.00254121	0.002335	0.0011128	0.00235556
Error Ratio	2.67×10^−5^	1.66×10^−5^	5.33×10^−5^	1.51×10^−5^	5.37×10^−5^

**Table 14:** Simulation parameters for support vector machine-based attack detection model.

Parameters	Standard IEEE System		
	Bus-14	Bus-39	Bus-57
Input Features	34	75	140
Total samples	1000 samples		
Train samples	750 samples		
Test samples	250 samples		
Kernel function	Radial Basis Function (RBF)		
Validation	4-Fold Cross Validation (CV)		
*σ*	45.19	45.19	45.19
*C*	1.005	1.005	1.005
Detection error	0.03090	0.03090	0.03090

**Discussion:** A smaller error ratio in DAE-based reconstruction is critical to extract a robust latent code. Table 2, Table 3, Table 4, Table 5, Table 6, Table 7, Table 8, Table 9, Table 10, Table 11, Table 12, Table 13 show that the SDIA affected data features are well-reconstructed with negligibly small reconstruction error and the error ratio. Furthermore, it can be seen from the tables that in all the corruption addition schemes i.e. EDAE, ZDEA, GDEA, the features are well reconstructed. However, the proposed EDAE outperforms the existing schemes and shows the best results in the reconstruction of the attacked feature values. Overall the features are reconstructed well as can be seen from the negligibly low value of the error and error ratio. These results confirm that the robust latent-space code representation has been obtained that can be employed as input to the SVM-based classification model.

**Figure 3:**
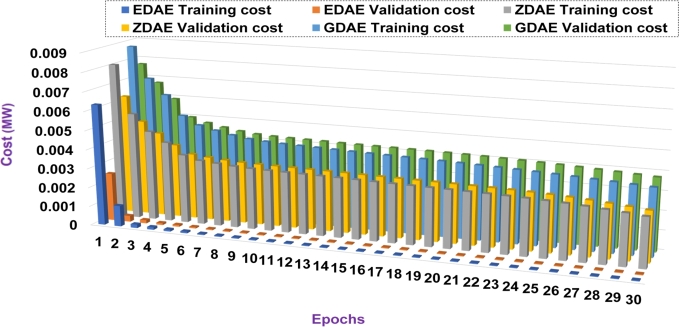
Training and validation costs for EDAE-, ZDAE-, and GDAE-based reconstruction schemes considering Nomadic Attack affecting 20% of SE-MF data features in Bus-39 system.

**Figure 4:**
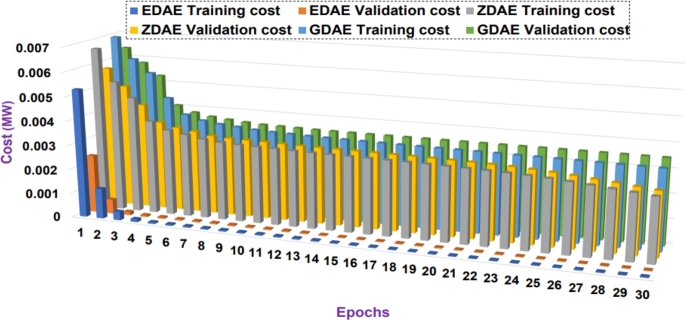
Training and validation costs for EDAE-, ZDAE-, and GDAE-based reconstruction schemes considering Nomadic Attack affecting 40% of SE-MF data features in Bus-39 system.

**Figure 5:**
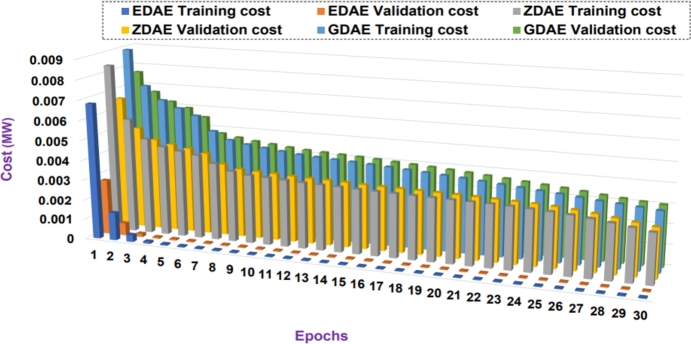
Training and validation costs for EDAE-, ZDAE-, and GDAE-based reconstruction schemes considering Nomadic Attack affecting 20% of SE-MF data features in Bus-14 system.

**Figure 6:**
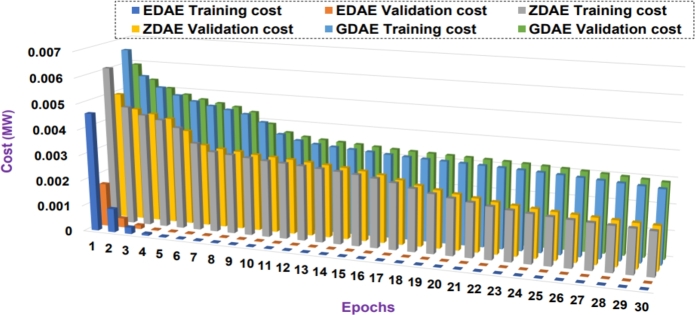
Training and validation costs for EDAE-, ZDAE-, and GDAE-based reconstruction schemes considering Nomadic Attack affecting 40% of SE-MF data features in Bus-14 system.

**Figure 7:**
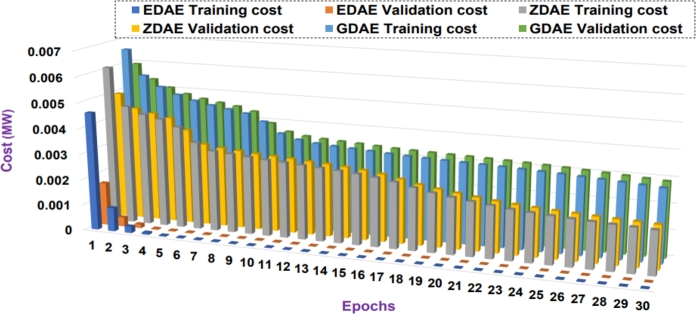
Training and validation costs for EDAE-, ZDAE-, and GDAE-based reconstruction schemes considering Static Attack affecting 20% of SE-MF data features in Bus-14 system.

**Figure 8:**
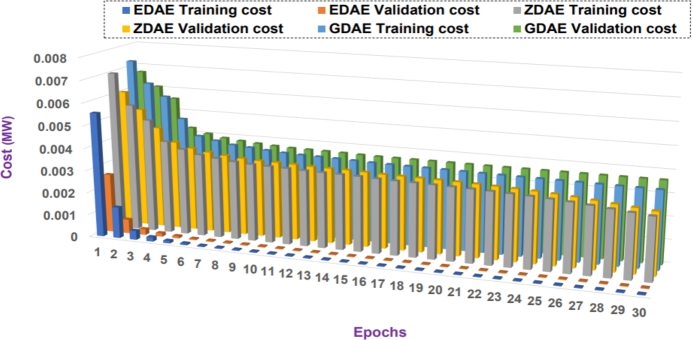
Training and validation costs for EDAE-, ZDAE-, and GDAE-based reconstruction schemes considering Static Attack affecting 40% of SE-MF data features in Bus-14 system.

Next we present the training and validation cost for the proposed DAE-based dimension reduction model. Figure 3, Figure 4, Figure 5, Figure 6, Figure 7, Figure 8 show the training and validation cost of the train data considering epochs for static- and nomadic-attacks launched in 20% and 40% of the data features, calculating the cost in megawatts.

**Discussion:** Considering the all test cases, it is clear from the Figure 3, Figure 4, Figure 5, Figure 6, Figure 7, Figure 8 that the proposed EDAE outperforms the existing techniques. It is evident that the reconstruction performance of zero-masking autoencoder surpasses that of the additive Gaussian noise AE scheme.

Broadly, we see that all schemes perform well to yield a robust code latent space that can be fed as input in SVM for SDIA detection.

Overall, the EDAE scheme outperforms the existing schemes with the lowest reconstruction error, error average ratio, and training/validation costs.

### 5.6 Performance evaluation of support vector machine-based detection model

Elementary performance evaluation parameters i.e., receiver operating characteristics (ROC)-curves, $F_{1}$-score and detection-accuracy are utilized in this work, and are presented in the subsequent subsections.

#### 5.6.1 Accuracy

One of the standard ways to assess the effectiveness of a classification model is to calculate accuracy which, is a single-number expression to validate the effectiveness of the proposed scheme. The calculation of accuracy, Acc, is performed in the following manner, utilizing equation (18):

(18) $$Acc=\left(\frac{T_{pos}+T_{neg}}{T_{pos}+F_{pos}+T_{neg}+F_{neg}}\right),$$

**Figure 9:**
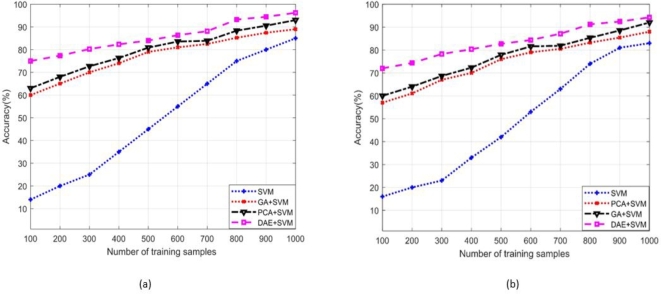
Accuracy comparison of the proposed DAE+SVM and existing schemes in the identification of (a) Static SDIA (b) Nomadic SDIA (standard IEEE 14-bus system).

**Figure 10:**
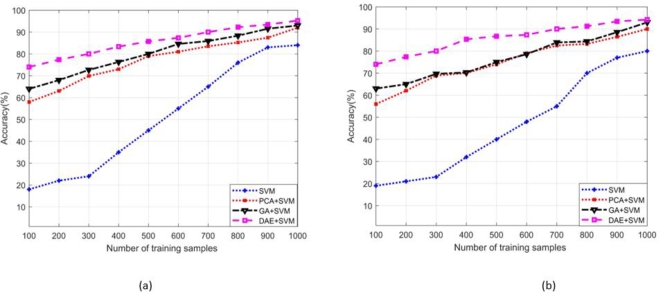
Accuracy comparison of the proposed DAE+SVM and existing schemes in the identification of (a) Static SDIA (b) Nomadic SDIA (standard IEEE 39-bus system).

The term “true positive” $\left(T_{pos}\right)$ refers to the data samples that the proposed scheme correctly identifies as positive examples, and these samples are indeed positive in reality. Similarly, true negatives $\left(T_{neg}\right)$ refer to the samples that the proposed model correctly identifies as negative data points, which indeed are negative. The $\left(F_{neg}\right)$ and $\left(F_{pos}\right)$ are the data points, erroneously detected by the classifier model in opposite category. Figure 9, Figure 10 depict the accuracy of the presented SDIA+SVM-based attack detection scheme across different standard IEEE bus systems (14-bus system is portrayed in Fig. 9 (a) and (b) for static and nomadic attacks, whereas 39-bus system is shown in Fig. 10 (a) and (b)) with a varying size of training dataset. We compare the effectiveness proposed scheme with existing dimension reduction schemes, such as genetic algorithm (GA)+SVM, a FS-based detection, and principal component analysis (PCA)+SVM, a FE-based detection. Simulation findings indicate that the proposed DAE+SVM with fine-tuned values of *C*, *σ*, and RBF kernel achieve higher accuracy than the other schemes and needs a few training samples. The GA+SVM and PCA+SVM result in reasonable SDIA detection accuracy, while affiliated performance enhances gradually with the increasing number of training samples. However, simple SVM (without DR) has a slow training speed and is hard to tune. The GA+SVM and PCA+SVM exhibit lesser accuracy compared to the proposed scheme. It is also observed that a considerably large number of training samples are needed in model training to achieve a suitable level of accuracy.

#### 5.6.2 Reciever operating characteristics curve

**Figure 11:**
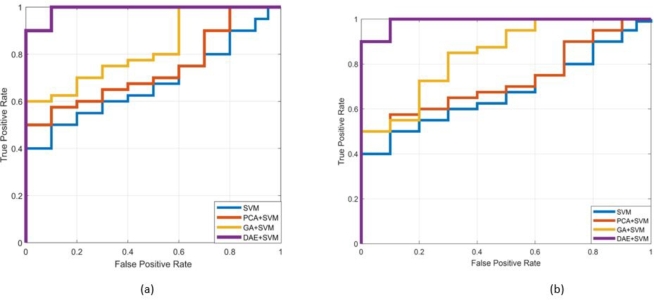
Reciever operating Curve for the proposed and existing schemes (a) Static SDIA (b) Nomadic SDIA (14-bus system).

**Figure 12:**
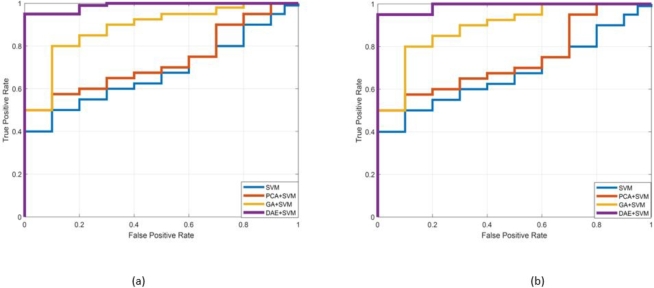
Reciever operating Curve for the proposed and existing schemes (a) Static SDIA (b) Nomadic SDIA (39-bus system).

Next, we proceed to present the receiver operating characteristic curves in order to demonstrate the efficacy of the proposed scheme. Figure 11, Figure 12 depict the ROC of the DAE+SVM scheme for the selected IEEE test cases for static and nomadic attacks scenarios on 20% and 40% affected SE-MF features. Fig. 11 (a), (b) and Fig. 12 (a), (b) are representing 14- and 39-bus systems respectively. The receiver operating characteristic curve is constructed by graphing the rate of false positives (FPR) against the rate of true positives (TPR). The false positive rate (FPR) refers to the likelihood of misclassifying unassailed data samples as assailed. In the detection scheme, it is a measure of specificity. On the contrary, TPR is a criterion of sensitivity and is defined as the likelihood of classifying assailed data samples as corrupted. Figure 11, Figure 12 show that the area under the curve for the proposed DAE-based scheme is roughly equal to one, substantiating its acceptable value efficiency. The area under the curve for the other schemes is comparatively low.

#### 5.6.3 $F_{1}$ score

Next, we compute the $F_{1}$-score, which is another measure for dissecting the classification accuracy. The $F_{1}$-score is deemed a yardsti for precision in categorization of the SE-MF data. The $F_{1}$ score is calculated utilizing equation (19):

(19) $$F_{1}=2\left(\frac{P_{r}\times R_{e}}{P_{r}+R_{e}}\right),$$

where precision $P_{r}$ is computed by using equation (20):

(20) $$P_{r}=\left(\frac{T_{pos}}{T_{pos}+F_{pos}}\right),$$

Predicted positives may contain both attacked and not-attacked data instances, but the classification model identifies all of them as positive. The term $R_{e}$ is commonly referred to as recall, given as in equation (21):

(21) $$R_{e}=\left(\frac{T_{pos}}{T_{pos}+F_{neg}}\right),$$

Typically, the $F_{1}$-score has a range up to 1. A classifier is regarded as more efficient if the value of $F_{1}$-score is closer to 1. A summary of $F_{1}$ score and accuracy is presented in Table 15.

**Table 15:** Comparison of SDIA detection Accuracy and *F*_1_ score for proposed and existing schemes.

Scheme	Standard IEEE System	Attack Type	Targeted Features	Accuracy (%)	*F*_1_ score
		Static	20%	92.915	0.920
		Static	40%	92.542	0.914
	14-bus	Nomadic	20%	91.662	0.907
		Nomadic	40%	91.003	0.453
DAE + SVM		Static	20%	94.842	0.934
		Static	40%	93.012	0.947
	39-bus	Nomadic	20%	92.763	0.903
		Nomadic	40%	92.672	0.940

		Static	20%	92.365	0.920
		Static	40%	91.321	0.914
	14-bus	Nomadic	20%	90.545	0.921
		Nomadic	40%	89.867	0.893
GA + SVM		Static	20%	93.842	0.934
		Static	40%	92.012	0.937
	39-bus	Nomadic	20%	92.763	0.923
		Nomadic	40%	91.672	0.909

		Static	20%	89.012	0.884
		Static	40%	88.652	0.880
	14-bus	Nomadic	20%	89.656	0.856
		Nomadic	20%	88.012	0.890
PCA + SVM		Static	20%	90.842	0.904
		Static	40%	90.412	0.900
	39-bus	Nomadic	20%	91.763	0.903
		Nomadic	40%	91.672	0.910

		Static	20%	85.012	0.8240
		Static	40%	82.012	0.850
	14-bus	Nomadic	20%	83.012	0.831
		Nomadic	40%	81.012	0.820
SVM		Static	20%	86.842	0.864
		Static	40%	84.412	0.840
	39-bus	Nomadic	20%	84.763	0.843
		Nomadic	40%	82.672	0.820

## 6 Conclusion

This paper presents a deep DAE-based scheme to address the curse of dimensionality that grows with the expanding size of the power system and pulls a robust latent space code from the SE-MF data set. The DAE apprehends more edifying latent-space imprints from the raw, attacked multivariate non-linear SE-MF dataset, and can gather a rugged representation. Next, the latent space representation code is fed an SVM model to identify SDIA, which is infused into the SE-MF data by the adversary acquainted with the power network hierarchy. The binary SVM axiomatically sketches a decision boundary that may acquire the most heightened geometric deviation between unassailed and assailed data points to categorize the data into assailed or normal data samples. We used standard IEEE 14-, 39-, and 57-bus test cases to simulate the SE-MF dataset. We compared the proposed scheme with existing approaches such as GA+SVM, PCA+SVM, and simple SVM. The results reveal that the suggested DAE+SVM-based approach (amidst fine-tuned values of C, *σ*, and RBF kernel), exhibits promising identification efficiency schemes under intermittent functioning conditions. Subsequently, the DAE+SVM performs better for SDIA detection in SG-based cyber-physical system communications networks.

## CRediT authorship contribution statement

**Anila Kousar:** Writing – original draft, Visualization, Validation, Software, Methodology, Formal analysis, Data curation. **Saeed Ahmed:** Writing – review & editing, Writing – original draft, Supervision, Project administration, Methodology, Formal analysis, Conceptualization. **Abdullah Altamimi:** Writing – review & editing, Methodology, Investigation. **Su Min Kim:** Writing – review & editing, Validation, Methodology, Investigation. **Zafar A. Khan:** Writing – review & editing, Supervision, Methodology, Conceptualization.

## Declaration of Competing Interest

The authors declare that they have no known competing financial interests or personal relationships that could have appeared to influence the work reported in this paper.

## Data Availability

Data included in the article is referenced while explaining the MATPOWER based load flow analysis.
